# High-Dose Cholecalciferol Booster Therapy is Associated with a Reduced Risk of Mortality in Patients with COVID-19: A Cross-Sectional Multi-Centre Observational Study

**DOI:** 10.3390/nu12123799

**Published:** 2020-12-11

**Authors:** Stephanie F. Ling, Eleanor Broad, Rebecca Murphy, Joseph M. Pappachan, Satveer Pardesi-Newton, Marie-France Kong, Edward B. Jude

**Affiliations:** 1 Tameside and Glossop Integrated Care NHS Foundation Trust, Fountain Street, Ashton-under-Lyne OL6 9RW, UK; stephanie.ling@manchester.ac.uk (S.F.L.); eleanor.broad@doctors.org.uk (E.B.); rebecca.murphy@tgh.nhs.uk (R.M.); 2 The University of Manchester, Oxford Road, Manchester M13 9PL, UK; pappachan.joseph@lthtr.nhs.uk; 3 Royal Preston Hospital, Lancashire Teaching Hospitals NHS Foundation Trust, Sharoe Green Lane, Fulwood, Preston PR2 9HT, UK; 4 Manchester Metropolitan University, All Saints Building, Oxford Road, Manchester M15 6BH, UK; 5 Leicester Royal Infirmary, University Hospitals of Leicester NHS Trust, Infirmary Square, Leicester LE1 5WW, UK; spardesi@hotmail.co.uk; 6 Leicester General Hospital, University Hospitals of Leicester NHS Trust, Gwendolen Road, Leicester LE5 4PW, UK; marie-france.kong@uhl.tr.nhs.uk

**Keywords:** SARS-CoV-2, COVID-19, vitamin D, mortality, cholecalciferol, 25-hydroxyvitamin D, vitamin D treatment, novel coronavirus 2019

## Abstract

The worldwide pandemic of 2019 novel coronavirus disease (COVID-19) has posed the most substantial and severe public health issue for several generations, and therapeutic options have not yet been optimised. Vitamin D (in its “parent” form, cholecalciferol) has been proposed in the pharmacological management of COVID-19 by various sources. We aimed to determine whether COVID-19 mortality was affected by serum 25-hydroxyvitamin D (25(OH)D) levels, vitamin D status, or cholecalciferol therapy, and to elucidate any other predictors of COVID-19 mortality. Patients hospitalised with COVID-19 were opportunistically recruited from three UK hospitals, and their data were collected retrospectively. Logistic regression was used to determine any relationships between COVID-19 mortality and potential predictors, including 25(OH)D levels and cholecalciferol booster therapy. A total of 986 participants with COVID-19 were studied, of whom 151 (16.0%) received cholecalciferol booster therapy. In the primary cohort of 444 patients, cholecalciferol booster therapy was associated with a reduced risk of COVID-19 mortality, following adjustment for potential confounders (OR_adj_ 0.13, 95% CI 0.05–0.35, *p* < 0.001). This finding was replicated in a validation cohort of 541 patients (OR_adj_ 0.38, 95% CI 0.17–0.84, *p* = 0.018). In this observational study, treatment with cholecalciferol booster therapy, regardless of baseline serum 25(OH)D levels, appears to be associated with a reduced risk of mortality in acute in-patients admitted with COVID-19. Further work with large population studies needs to be carried out to determine adequate serum 25(OH)D levels, as well as multi-dose clinical trials of cholecalciferol therapy to assess maximum efficacy.

## 1. Introduction

The worldwide pandemic of severe acute respiratory syndrome coronavirus 2 (SARS-CoV-2) has presented the largest global public health problem in several generations. A vast amount of rapid research has taken place to find an effective therapeutic agent to manage 2019 novel coronavirus disease (COVID-19), which is caused by SARS-CoV-2. Vitamin D (which will henceforth be referred to as cholecalciferol, i.e., in its “parent”, therapeutic form, vitamin D_3_) has been proposed as a potential adjuvant to therapy for COVID-19 in a number of recent studies [1,2,3,4], as cholecalciferol has previously been suggested to have an antiviral effect [5]. Serum 25-hydroxyvitamin D (25(OH)D) is the measurable metabolite of cholecalciferol that is used to determine an individual’s vitamin D status. Other reviews have suggested that replete vitamin D status (serum 25(OH)D) >50 nmol/L) may be important in preventing severe manifestations of COVID-19 [6,7]. Other sources have proposed the interleukin (IL)-6 inhibitor tocilizumab as a potential treatment for COVID-19, and due to its modulator effect on IL-6, cholecalciferol has again been postulated as a potential therapeutic option [8].

A UK-based study by Panagiotou et al. found that low serum 25(OH)D levels in COVID-19 in-patients were associated with a more severe disease course [9], but this small study of 134 patients only looked at serum levels and not concurrent cholecalciferol therapy. Furthermore, two meta-analyses have identified low serum 25(OH)D levels as a potential predictor of more severe COVID-19 disease outcomes [10,11], again without addressing any effect of treatment. To our knowledge, no observational study has addressed the effect of cholecalciferol therapy on outcomes on an individual-level basis, and very few studies exist regarding serum 25(OH)D levels and the risk of COVID-19 mortality following in-patient admission.

Therefore, the primary research question of this study was to determine whether serum 25(OH)D levels and/or deficient vitamin D status affect mortality in COVID-19 infection. Our secondary objective was to determine whether any other patient characteristics were associated with COVID-19 mortality. To reach these objectives, we carried out a retrospective multi-centre cross-sectional observational study.

## 2. Materials and Methods

### 2.1. Participants

For the primary analysis, patients were opportunistically recruited from an acute hospital trust in the UK, namely Tameside and Glossop NHS Foundation Trust (recruitment from Tameside General Hospital). Tameside General Hospital is a district general hospital serving a population of 250,000 people living in both urban and rural areas in Greater Manchester and Derbyshire. For the validation analysis, patients were recruited from two additional acute hospital Trusts, also in the UK: Lancashire Teaching Hospitals NHS Foundation Trust (recruitment from Royal Preston Hospital), and University Hospitals of Leicester (UHL) NHS Trust (comprising of Glenfield Hospital, Leicester General Hospital, and Leicester Royal Infirmary). Patients were recruited from all three sites comprising UHL, and samples were processed at the same laboratory, based at Leicester Royal Infirmary. Royal Preston Hospital serves a population of 370,000 people living in both urban and rural areas in Lancashire and South Cumbria. The UHL NHS Trust serves 1,000,000 patients across Leicestershire; Leicester Royal Infirmary has the county’s only emergency department.

Ethical approval was granted by the Health and Care Research Wales Research Ethics Committee (IRAS number 285337). The study was also registered on Clinicaltrials.gov (reference number NCT04386044), prior to commencement. Although this was not an interventional study, we registered it on an open-access database for transparency. Informed written consent was not required as this was rapid COVID-19 research carried out prior to March 2021, as per Health Research Authority (HRA) guidance in the UK [12]. The UK Government announced emergency arrangements for the use of confidential patient information without consent for COVID-19 research purposes, and this was included in our ethical application, which was approved following fast tracking.

Patients recruited were admitted between 27th January 2020 and 5th August 2020, and data were collected retrospectively between 26th June 2020 and 7th August 2020. Although the first case of COVID-19 reported in the UK was on approximately 29th January 2020 and the first patient recruited to the study was admitted on 27th January 2020, this patient developed COVID-19 as an in-patient following admission for a different condition. Data collection commenced as soon as ethical approval had been obtained, and stopped once the investigators felt that the first peak of COVID-19 admissions had passed.

In-patients with a clinical diagnosis of COVID-19 identified by clinical coding (emergency use ICD code U07·1, COVID-19 confirmed by laboratory testing, and code U07·2, COVID-19 diagnosis where laboratory confirmation is inconclusive or not available [13]) were all included in the study. Laboratory testing for COVID-19 was carried out using throat ± nasal swab, and samples were tested for SARS-CoV-2 viral RNA following amplification using real-time PCR. A clinical diagnosis of COVID-19 was made if laboratory testing was negative, but patients had symptoms and signs suggestive of SARS-CoV-2 infection, such as persistent dry cough, low oxygen saturations (SpO_2_), fever, dyspnoea, bilateral interstitial infiltrates on a chest radiograph or computed tomography (CT) scan, etc. Patients were excluded if they were younger than 18 years of age or if the final clinical diagnosis was not COVID-19. Demographic and clinical data were obtained from the hospital admission associated with the diagnosis of COVID-19 using a combination of electronic patient records (EPR), hard-copy patient records, and hospital laboratory data.

The primary outcome measure, COVID-19 mortality, included deaths in hospital, and deaths following admission recorded during the data collection period, e.g., following transfer or discharge. Potential predictors of mortality consisted of: baseline serum 25(OH)D levels, deficient vitamin D status (serum 25(OH)D < 25 nmol/L), treatment with cholecalciferol using high-dose booster therapy (approximately ≥ 280,000 IU in a time period of up to 7 weeks [14]), age, sex, non-Caucasian ethnicity, hospital-acquired COVID-19, clinical parameters on admission (SpO_2_, C-reactive protein (CRP), creatinine, random glucose), progression to either continuous positive airways pressure (CPAP) therapy or invasive mechanical ventilation (IMV), length of stay, and common medical comorbidities of interest (including both type 1 and type 2 diabetes), as listed by clinicians in patient medical records. A full list of variables measured and how they were obtained is listed in Table S1. Patients received cholecalciferol booster therapy if they were recognised as being either vitamin D insufficient (serum 25(OH)D 25–50 nmol/L) or deficient as part of routine clinical care. Hospital-acquired COVID-19 was defined as: (i) if a patient had been admitted with a different acute condition and had gone on to develop COVID-19 whilst an in-patient; or (ii) if a patient had been re-admitted within the 14-day incubation period and the second admission was for COVID-19. All laboratory measurements (including serum 25(OH)D levels) were carried out as part of routine clinical care of patients during their acute in-patient admissions.

### 2.2. Measurement of Serum 25(OH)D Levels

Serum 25(OH)D was measured using the UniCel Dxl 800 Access Immunoassay System (Beckman Coulter Life Sciences, Indianapolis, IN, USA) at Tameside General Hospital, the cobas e 801 analytical unit (Roche, Basel, Switzerland) at Royal Preston Hospital, and the ADVIA Centaur XPT Immunoassay System (Siemens Healthineers, Erlangen, Germany) at UHL. As serum 25(OH)D measurement was not part of an established care protocol at any participating site, it was at the discretion of physicians caring for patients whether to order this test. Hence, some participants still have missing values, as measurements were carried out as part of routine clinical care, and not specifically for participation in this study. Serum 25(OH)D measurements were ordered if patients were deemed to be at risk of insufficiency or deficiency (e.g., non-Caucasian ethnicity, elderly, lack of exposure to sunlight). In addition, as evidence emerged over the course of the COVID-19 pandemic regarding serum 25(OH)D and disease outcomes, patients with suspected or confirmed COVID-19 were also included on the list of patients at risk of insufficiency or deficiency. Serum 25(OH)D levels up to 12 weeks prior to admission with acute COVID-19 were included if not measured during each participant’s in-patient stay, in order to increase power. This time period was set to mitigate for seasonal variation; we chose not to include older serum 25(OH)D measurements for this reason.

The clinical laboratories at Tameside General Hospital participate in the Randox International Quality Assessment Scheme (RIQAS) [15] in order to ensure external quality assessment of all assays, including 25(OH)D. The clinical laboratories at Royal Preston Hospital participate in the Vitamin D External Quality Assessment Scheme (DEQAS) [16] to ensure analytical reliability of its 25(OH)D assays. The clinical laboratories at UHL participate in DEQAS, as well as the UK National External Quality Assurance Scheme (NEQAS) [17] for external quality assessment of all assays.

### 2.3. Statistical Methods

The Wilcoxon rank–sum test was used to determine whether 25(OH)D assays were significantly different by centre, due to non-parametric distributions of this variable. In both the primary and validation cohorts, logistic regression was used to analyse predictor variables for potential associations with COVID-19 mortality, with adjustment for the following variables, which are known to be associated with COVID-19 mortality: age, sex, obesity, non-Caucasian ethnicity, and diabetes (types 1 and 2 combined). Median values were used to convert linear variables to binary high/low variables, as none of these variables had a parametric distribution. Binary variables were created separately for both primary and validation cohorts, as cut-off values were slightly different between the two independent populations. Variables with significant associations were placed into multivariate logistic models to adjust for any potential interactions between predictors and potential confounders. In the validation cohort, analysis was additionally adjusted for the centre from which participants were recruited. Missing values were treated as missing data, and values were not imputed, because of the nature of the clinical data collected. All analysis was carried out using Stata (StataCorp LLC, College Station, TX, USA), version 14.0.

## 3. Results

### 3.1. Cohort Characteristics

A total of 444 participants were included from Tameside General Hospital for primary analysis. Full summary statistics of this cohort are detailed in Table 1. The median age of participants was 74 (interquartile range, IQR, 63, 83) and 199 participants were female (44.9%). The median serum 25(OH)D level was 31.2 (IQR 18.9, 54.9), in a total of 230 participants with available serum 25(OH)D levels. The mean serum 25(OH)D level was 40.2 nmol/L, with a SD of 30.5. Serum 25(OH)D values ranged between 1.1 and 165.2 nmol/L. 63 (27.4%) participants were vitamin D replete, 80 (34.8%) were in the insufficient range, and 87 (37.8%) were deficient. Of the 329 participants with information available regarding cholecalciferol prescription, 73 (17.1%) were on high-dose booster therapy; the various regimens are detailed in Table 2.

A total of 542 participants were recruited to the validation cohort from Royal Preston Hospital and UHL combined (231 and 311 participants, respectively). Serum 25(OH)D levels were significantly different between patients recruited from Tameside General Hospital and both Royal Preston Hospital (*p* < 0.001) and UHL (*p* < 0.001). However, there was no significant difference in serum 25(OH)D levels between Royal Preston Hospital and UHL (*p* = 0.1677), so participants recruited from these sites have been pooled to form a validation cohort. Condensed summary statistics of the validation cohort are detailed in Table 3; detailed summary statistics of both centres that comprise the validation cohort are available in Tables S2 and S3.

The median serum 25(OH)D level at Royal Preston Hospital was 45 (IQR 27, 72). The mean serum 25(OH)D level was 51.9 nmol/L, with a SD of 31.2. Serum 25(OH)D levels ranged between 13 and 126 nmol/L. 106 (45.9%) patients were vitamin D replete, 73 (31.6%) were in the insufficient range, and 52 (22.5%) were deficient. The median serum 25(OH)D level at UHL was 43 (IQR 27, 60). The mean serum 25(OH)D level was 46.4 nmol/L, with a SD of 23.8. Serum 25(OH)D levels ranged between 15 and 113 nmol/L. 110 (37.4%) patients were replete, 125 (42.5%) were in the insufficient range, and 59 (20.1%) were deficient. 59 participants out of 227 (26.0%) with information available regarding cholecalciferol prescription were on high-dose booster therapy at Royal Preston Hospital, and 19/296 (6.4%) at UHL; the various regimens are detailed in Table 4. Differences in the numbers of participants prescribed cholecalciferol booster therapy at different centres are detailed in Table 5.

### 3.2. Predictors of COVID-19 Mortality in the Primary Cohort

Two predictors were associated with COVID-19 mortality in univariate analysis, and are detailed in Table 6. Following adjustment for potential confounders, these consisted of: admission CRP > 82 mg/L (OR_adj_ 1.3, 95% CI 1·04–2.55, *p* = 0.026) and admission creatinine > 84 μmol/L (OR_adj_ 1.63, 95% CI 1.04–2.55, *p* = 0.032). In addition, three predictors were associated with reduced risk of mortality from COVID-19: age > 74 years (OR_adj_ 0.48, 95% CI 0.24–0.97, *p* = 0.040), treatment with cholecalciferol booster therapy (OR_adj_ 0.25, 95% CI 0.12–0.49), *p* < 0.001), and a diagnosis of asthma (OR_adj_ 0.31, 95% CI 0.13–0.71, *p* = 0.006). These associations were unchanged when sub-analysis was carried out in only patients with a positive SARS-CoV-2 swab (Table S4). Sub-analysis stratified by vitamin D status is presented in Tables S5–S7.

**Figure 1:**
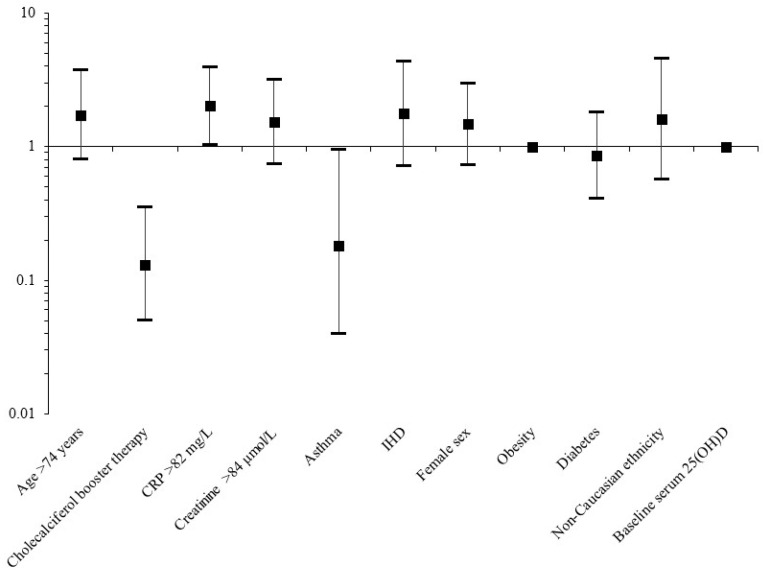
Predictors associated with mortality from COVID-19 following multivariate analysis in the primary cohort.

Significant predictors were then placed in a multivariate model, along with the potential confounding variables of sex, obesity, non-Caucasian ethnicity, diabetes, and baseline serum 25(OH)D levels (Table 7, Figure 1). Following adjustment in the multivariate model, only CRP > 82 mg/L on admission remained significantly associated with mortality from COVID-19 (OR_adj_ 2.01, 95% CI 1.03–3.91, *p* = 0.040). However, two variables remained protective of mortality from COVID-19: treatment with high-dose cholecalciferol booster therapy (OR_adj_ 0.13, 95% CI 0.05–0.35, *p* < 0.001) and asthma (OR_adj_ 0.18, 95% CI 0.04–0.94, *p* = 0.042). Patients with asthma were significantly younger, with fewer patients in the age > 74 years category (*p* < 0.001). Again, these associations were unchanged when sub-analysis was carried out in only patients with a positive SARS-CoV-2 swab (Table S8).

In the primary cohort, serum 25(OH)D levels were still not associated with COVID-19 mortality when patients treated with high-dose booster therapy were removed from the analysis (OR 1.00, 95% CI 0.99–1.01, *p* = 0.533). This finding persisted following adjustment for the above potential confounding variables (OR_adj_ 1.00, 95% CI 0.99–1.01, *p* = 0.512).

### 3.3. Validation of COVID-19 Mortality Associations in the Validation Cohort

In comparison with the primary cohort, cut-off values for binary variables differ slightly in the validation cohort, as these were based on median values for each individual cohort, and not for the total study population. Several predictors were associated with COVID-19 mortality in the validation cohort, following adjustment for potential confounders (Table 8): age > 73 years (OR_adj_ 3.26, 95% CI 1.99–5.36), *p* < 0.001), vitamin D deficiency (OR_adj_ 1.87, 95% CI 1.09–3.27, *p* = 0.024), admission SpO_2_ < 96% (OR_adj_ 1.88, 95% CI 1.21–2.93, *p* = 0.005), admission CRP > 73 mg/L (OR_adj_ 1.87, 95% CI 1.19–2.95, *p* = 0.007), admission creatinine > 83 μmol/L (OR_adj_ 2.10, 95% CI 1.29–3.42, *p* = 0.003), CPAP therapy (OR_adj_ 4.48, 95% CI 2.17–9.33, *p* < 0.001), admission glucose > 6.9 mmol/L (OR_adj_ 1.69, 95% CI 1.02–2.80, *p* = 0.040), and a diagnosis of ischaemic heart disease, IHD (OR_adj_ 1.85, 95% CI 1.07–3.18, *p* = 0.027). Only one predictor was associated with reduced risk of mortality—treatment with cholecalciferol booster therapy (OR_adj_ 0.40, 95% CI 0.20–0.82, *p* = 0.012). These associations were unchanged when sub-analysis was carried out in only patients with a positive SARS-CoV-2 swab (Table S9). A sub-analysis, stratified by vitamin D status, is presented in Tables S10–S12.

**Figure 2:**
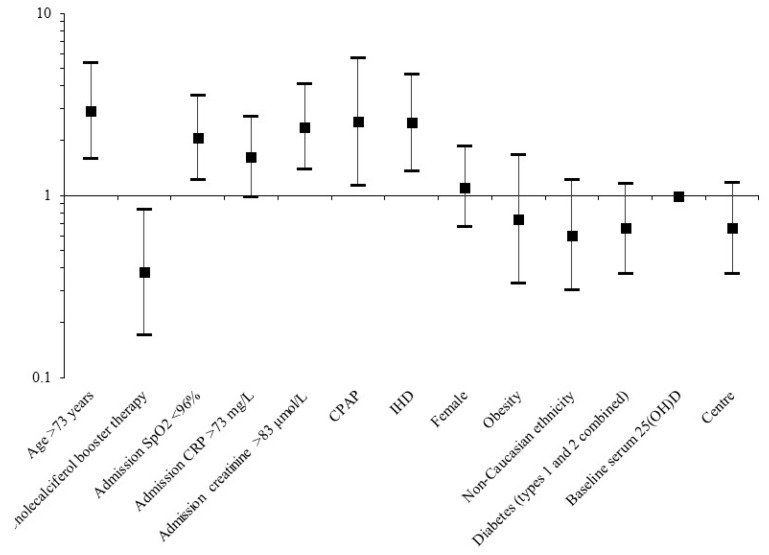
Predictors associated with death from COVID-19 following multivariate analysis in the validation cohort.

Significant predictors were then placed in a multivariate model, along with potential confounders (Table 9, Figure 2). Instead of vitamin D deficient status, baseline serum 25(OH)D levels were instead placed in the model, in order to replicate analysis from the primary cohort. Multivariate analysis was also adjusted for the centre from which participants were recruited. Of the above predictors of mortality, the following retained significance: age > 73 years (OR_adj_ 2.90, 95% CI 1.58–5.33, *p* = 0.001), admission SpO_2_ < 96% (OR_adj_ 2.08, 95% CI 1.22–3.53, *p* = 0.018), admission creatinine > 83 μmol/L (OR_adj_ 2.38, 95% CI 1.39–4.07, *p* = 0.002), CPAP therapy (OR_adj_ 2.54, 95% CI 1.13–5.69, *p* = 0.023), and a diagnosis of IHD (OR_adj_ 2.51, 95% CI 1.36–4.64, *p* = 0.003). Treatment with cholecalciferol booster therapy remained significantly associated with reduced risk of mortality (OR_adj_ 0.38, 95% CI 0.17–0.84, *p* = 0.018), which replicated findings from the primary cohort. Again, these associations were unchanged when sub-analysis was carried out in only patients with a positive SARS-CoV-2 swab (Table S13).

Finally, our finding that serum 25(OH)D levels were not associated with COVID-19 mortality when patients receiving high-dose booster therapy were removed from analysis replicated in the validation cohort (OR 1.00, 95% CI 0.99–1.01, *p* = 0.802). Again, this finding persisted following adjustment for potential confounding variables (OR_adj_ 0.99, 95% CI 0.98–1.00, *p* = 0.122).

## 4. Discussion

To our knowledge, this is the largest observational study of hospital in-patients with COVID-19 to examine any potential associations between the treatment of the acute infection and vitamin D status, and cholecalciferol treatment. Serum 25(OH)D levels were not associated with COVID-19 mortality in both primary and validation cohorts, and deficient vitamin D status was not associated with COVID-19 mortality in the primary cohort. However, treatment with cholecalciferol appeared to be protective against mortality, regardless of baseline serum 25(OH)D levels, and this replicated across both cohorts.

Our findings regarding 25(OH)D levels appear to fit with a study utilising participants from the UK Biobank, which found no association between serum 25(OH)D levels and risk of COVID-19 infection [18]. The UK Biobank study looked at 348,598 participants, of whom, 449 had a confirmed diagnosis of COVID-19 as defined by a positive laboratory test for SARS-CoV-2 (only 0.13% of study population). However, it is likely that the COVID-19 cases from that study were managed in a mixture of hospitals and the community, and serum 25(OH)D was measured between 2006 and 2010, and not contemporaneously with COVID-19 infection 10–14 years after recruitment to the UK Biobank. Our study adds extra information regarding patients who, by their nature, have more severe disease, as they have been hospitalised. Additionally, our study provides information on 25(OH)D levels as close to acute COVID-19 infection as possible (as opposed to up to 14 years before contracting COVID-19, as in the UK Biobank study), giving a more accurate picture of any interactions; we imposed a limit of 12 weeks on 25(OH)D levels prior to admission to mitigate for seasonal variation, whilst also including as many measured 25(OH)D levels as possible to maximise power.

Rhodes et al. suggest that countries at a latitude above 35 degrees North have experienced increased mortality from COVID-19, suggesting a potential role of cholecalciferol therapy in COVID-19 treatment [19], but our findings do not implicate 25(OH)D levels in the role of increasing mortality rates in these countries. In this editorial, statistics of COVID-19 mortality by country are compared to latitude, and because countries above 35 degrees North are shown to have increased mortality per million population compared to those south of this latitude, the authors postulate that vitamin D insufficiency may be a contributing factor, due to a lack of sunlight during the winter months. This is not an experimental epidemiological study and does not include data on 25(OH)D levels or vitamin D status, in comparison to our study, which includes both.

Two independent studies from Israel [20] and the USA [21] found that deficient vitamin D status was associated with an increased risk of COVID-19. These studies differ from our own because they only studied the risk of SARS-CoV-2 infection, and not of mortality. Our study also differs in that it only includes hospitalised patients, who are already known to have developed COVID-19, so we were unable to assess whether vitamin D status was associated with the risk of SARS-CoV-2 infection, due to the nature of our study population. Our findings appear to differ from some of these other studies, but this could be due to power issues or a differing population. Given the emerging nature of this research, large meta-analyses will be required in the future when more data are available from multiple international sites.

Interestingly, treatment with high-dose cholecalciferol booster therapy was associated with a reduced odds of death, even following adjustment for baseline serum 25(OH)D levels [14]. This could be due to a number of reasons. Firstly, it may be because it is not clear what an adequate amount of cholecalciferol supplementation is required to maintain immune health. UK guidance is that serum 25(OH)D levels > 25 nmol/L are required to maintain musculoskeletal health [22], but this is within the range for deficiency [14] and does not take into account cholecalciferol’s role outside of musculoskeletal health. It is possible that serum 25(OH)D levels might need to be higher than the recommended range in order to provide protection from more severe COVID-19 outcomes. An alternative hypothesis might be that not all patients had 25(OH)D levels measured during admission (755/986 participants), and the patients that had levels measured and acted on may have received overall more intensive treatment, resulting in better outcomes, i.e., a proportion of patients had 25(OH)D levels measured, but may not have been prescribed replacement cholecalciferol treatment, for unknown clinical reasons. From the level of data collected, it is not clear what the mechanisms are behind our findings.

It may seem paradoxical that whilst treatment with high-dose cholecalciferol reduces the risk of COVID-19 mortality, neither baseline serum 25(OH)D levels nor vitamin D deficient status had an effect on mortality risk in the primary cohort. Vitamin D deficient status was associated with increased mortality in the validation cohort following adjustment for potential confounders, so the lack of association in the primary cohort may have been due to superior power to detect the association in the validation cohort.

However, our findings may have been due to the concept of the “personal vitamin D response” put forward by Carlberg et al. [23]. Two studies in independent populations (one in elderly pre-diabetic patients [24], and another in young, healthy subjects [25]) demonstrated changes in mRNA expression following administration of cholecalciferol. A range of clinical and biochemical parameters were also tested, but individual participants appeared to express these parameters differently from one another within the same studies. The authors classified participants as high/moderate/low responders to cholecalciferol based on the number of altered parameters that they exhibited following cholecalciferol administration, and they found that up to 25% of participants were low responders [23].

Given that our study was only carried out on hospital in-patients (i.e., those with the most severe clinical manifestations of COVID-19), if Carlberg et al.’s hypothesis [23] holds true, it is possible that in our study population there could be patients who are low responders to cholecalciferol who have seemingly adequate levels. Conversely, a high responder with seemingly deficient serum 25(OH)D levels could have only mild or no COVID-19 symptoms and not require hospitalisation, as they are able to utilise much lower 25(OH)D levels to better effect. Therefore, by analysing only patients who have been hospitalised, our population may include vitamin D high responders with low serum 25(OH)D levels, as well as vitamin D low responders with sufficient serum 25(OH)D levels. Statistically, these participants could cancel out the effect of the other, explaining why no association is seen between serum 25(OH)D levels and mortality. Once patients are treated with a high enough dose of cholecalciferol, we can then see an effect on reduction in mortality risk, as the dose will be high enough to overcome low vitamin D responder status. However, this is merely supposition, and large-scale clinical studies would need to be carried out in order to validate this theory.

Nonetheless, cholecalciferol as a potential therapeutic option for COVID-19 is an attractive prospect, given its wide availability and low cost, particularly in developing nations, as well as its relatively safe side-effect profile, in conjunction with regular monitoring of serum levels and serum adjusted calcium. Small-scale clinical trials are already beginning to populate the literature. In a pilot study of 76 patients hospitalised with COVID-19 in a single Spanish centre, Entrenas Castillo et al. found that fewer patients who were treated with calcifediol (hydroxylated cholecalciferol, also known as 25-hydroxyvitamin D_3_) were admitted to the intensive therapy unit when compared to controls [26]. Interestingly, 25(OH)D levels were not available on participants in this trial, although it is now being scaled up to include 15 hospitals across Spain. In addition, Rastogi et al. have recently completed a randomised placebo-controlled trial of high-dose cholecalciferol therapy (60,000 IU cholecalciferol for 7 days) in 40 SARS-CoV-2-positive patients who were asymptomatic or only mildly symptomatic, based in a single centre in India [27]. A greater proportion of patients in the treatment group achieved SARS-CoV-2 negativity at the end of the 14-day study period, and fibrinogen levels (which were used as a biomarker of inflammatory response) were significantly lower in the treatment arm. These studies are making headway in ascertaining the role of cholecalciferol/calcifediol therapy both at the pre-hospital and post-hospitalisation stage, and further work must be done to ascertain optimum dosage regimens and whether prophylactic therapy is of benefit in the setting of COVID-19 infection.

It is unsurprising that predictors such as age > 73 years, IHD and baseline creatinine > 83 μmol/L are associated with increased risk of death from COVID-19 (as seen in the multivariate analysis in the validation cohort), as these are likely to represent patients with a poorer baseline of health with less physiological reserve to adequately cope with acute COVID-19 infection. These patients are also in a group at risk of vitamin D insufficiency or deficiency. The June 2020 report from the Office of National Statistics, ONS (covering England and Wales, where this study population was recruited from), shows an exponential rise in age-specific mortality rates as age increases [28], and this fits with our data. Furthermore, a large multi-centre study in Italy found that older age, chronic kidney disease and coronary artery disease were more common in patients who died [29], and our findings agree with this. We found the opposite with the age > 74 years variable in the primary cohort following adjustment for multiple confounders, but this is likely to be a type 1 error, due to the smaller sample size (and hence, reduced power) of the primary cohort. We do not feel that this weakens any associations, however, as the one finding that has replicated across both cohorts is that high-dose cholecalciferol booster therapy is protective of COVID-19 mortality.

This study’s strengths lie in its recruitment of almost 1000 acute COVID-19 hospital in-patients from three separate centres, with a high proportion of patients with available serum 25(OH)D levels. It is also one of the largest observational studies, to date, to assess the benefit of cholecalciferol therapy in reducing the risk of COVID-19 mortality. The fact that our findings replicate across participants recruited from independent study populations strengthens the association between high-dose cholecalciferol booster therapy and a reduced risk of COVID-19 mortality. Our study population is potentially generalisable to the rest of the UK, with similar demographics across centres compared with the ONS figures on COVID-19 admissions that were most up to date at the time that recruitment closed [28]. Patients were recruited from throughout the pandemic, so our population reflects changing treatment recommendations as the pandemic evolved and evidence increased.

There are potential limitations to our study. For instance, not all patients had serum 25(OH)D levels available, so power may have been improved with more values. In addition, while results are potentially generalisable to the UK, results would need to be replicated in different international populations to assess transferability of findings globally. Another limitation lies in the fact that, although we imposed a 12-week time limit on pre-admission serum 25(OH)D measurements, the half-life of this form of vitamin D is 15–25 days, less than our 84-day limit, which we imposed to mitigate for seasonal variation whilst also aiming to include as many values as possible. However, only 14/227 participants (6.2%) with available serum 25(OH)D levels had measurements over 25 days pre-admission, with those 14 participants’ values having a median of 51 days (IQR 41, 63) prior to admission. Finally, due to the cross-sectional nature of this study, we are unable to ascertain cause and effect between associations, and we do not have a mechanistic understanding of our findings as yet. A longitudinal analysis of outcomes must be carried out in the future to determine any long-term sequelae of deficient vitamin D status during acute COVID-19 infection. There is also the potential for studies in whole blood and/or tissue to understand the mechanisms behind vitamin D status and COVID-19 severity.

## 5. Conclusions

In conclusion, high-dose cholecalciferol booster therapy, regardless of baseline serum 25(OH)D levels, appears to be associated with a reduced risk of mortality in acute in-patients admitted with COVID-19. This suggests that further work should be carried out to determine what an adequate serum level of 25(OH)D might be from large-scale population studies, and paves the way for future clinical trials of cholecalciferol therapy, at multiple doses in order to assess maximum efficacy. This inexpensive and widely available treatment could have positive implications for the management of COVID-19 worldwide, particularly in developing nations.

## Figures and Tables

**Table 1:** Participant characteristics of the primary study population.

Participant Characteristics	Tameside General Hospital (*n* = 444)	Number of Participants with Available Data
Age (years), median [IQR]	74 [63, 83]	444
Female, *n* (%)	199 (44.8)	444
Ethnicity, *n* (%)		444
Caucasian	386 (86.9)	
South Asian	43 (9.7)	
East Asian	2 (0.5)	
African Caribbean	5 (1.1)	
Other	8 (1.8)	
All non-Caucasian combined	58 (13.0)	
Serum 25(OH)D level (nmol/L), median [IQR]	31.2 [18.9, 54.9]	230
Vitamin D status, *n* (%)		230
Replete (>50 nmol/L)	63 (27.4)	
Insufficient (25–50 nmol/L)	80 (34.8)	
Deficient (<25 nmol/L)	87 (37.8)	
Received cholecalciferol treatment, *n* (%)	164 (50.3)	326
Maintenance treatment, *n* (% of patients on treatment)	88 (53.7)	
High-dose booster treatment, *n* (%)	73 (17.3)	
Positive SARS-NCoV2 swab, *n* (%)	403 (90.8)	444
Death, n (%)	177 (40.9)	433 *
Hospital-acquired, *n* (%)	126 (33.7)	374
Pulmonary embolism (PE) during admission, *n* (%)	15 (3.5)	435
Oxygen saturation (SpO_2_) on admission, median [IQR]	96 [94, 98]	356 ^†^
C-reactive protein (CRP) on admission (mg/L), median [IQR]	82 [30, 165]	435
Creatinine on admission (μmol/L), median [IQR]	84 [63, 119]	441
Adjusted calcium on admission (mmol/L), median [IQR]	2.3 [2.2, 2.4]	140
Random glucose on admission (mmol/L), median [IQR]	6.7 [5.7, 8.5]	428
Received low-flow oxygen (<10 L/min), *n* (%)	222 (72.5)	306
Received high-flow oxygen (≥10 L/min), *n* (%)	95 (40.4)	235
Received continuous positive airways pressure (CPAP), *n* (%)	41 (10.9)	375
Received invasive mechanical ventilation (IMV), *n* (%)	30 (7.6)	394
Discharged, *n* (%)	234 (52.7)	444
Length of stay (days), median [IQR]	10 [5, 19]	418 *
Diabetes mellitus (both types 1 and 2 combined), *n* (%)	129 (29.1)	444
Chronic obstructive pulmonary disease (COPD), *n* (%)	100 (22.5)	444
Asthma, *n* (%)	52 (11.7)	444
Ischaemic heart disease (IHD), *n* (%)	73 (16.4)	444
Current or previous acute coronary syndrome (ACS), *n* (%)	48 (10.8)	444
Heart failure, *n* (%)	54 (12.2)	444
Hypertension, *n* (%)	197 (44.4)	444
Current or previous transient ischaemic attack (TIA) or stroke, *n* (%)	40 (9.0)	444
Dementia, *n* (%)	59 (13.3)	444
Obesity, *n* (%)	20 (4.5)	444
Malignancy of solid organ, *n* (%)	71 (16.0)	444
Malignancy of skin, *n* (%)	8 (1.8)	444
Haematological malignancy, *n* (%)	8 (1.8)	444
Solid organ transplant, *n* (%)	4 (0.9)	444
Inflammatory arthritis, *n* (%)	16 (3.6)	444
Inflammatory bowel disease, *n* (%)	5 (1.1)	444

* Mortality and length of stay data are missing on patients recruited who were current in-patients at the time of data collection. ^†^ Admission SpO_2_ data missing as entered on paper record, not electronic record. Comorbidities were listed if a patient had ever been diagnosed with a condition, not just during acute admission during data entry period, e.g., malignancy of solid organ.

**Table 2:** Numbers of participants from the primary cohort on different regimens of cholecalciferol booster therapy.

Cholecalciferol Regimen	Number of Participants (%)
40,000 IU daily for 7 days	2 (2.7)
20,000 IU daily for 14 days	1 (1.4)
50,000 IU weekly	2 (2.7)
40,000 IU weekly	35 (47.9)
20,000 IU twice weekly	21 (28.8)
20,000 IU weekly	8 (11.0)
20,000 IU every two weeks	4 (5.5)

Apart from the participants receiving a booster regimen over seven or 14 days, participants received cholecalciferol booster therapy for a maximum of 7 weeks.

**Table 3:** Condensed participant characteristics of the validation cohort.

Participant Characteristics	Royal Preston Hospital (*n* = 231)	University Hospitals of Leicester (UHL) (*n* = 309)
Age (years), median [IQR]	76 [61, 84]	70 [56, 84]
	(*n* = 231)	(*n* = 309)
Female, *n* (%)	106 (42.2)	145 (57.8)
	(*n* = 231)	(*n* = 309)
Ethnicity, *n* (%)		
Caucasian	213 (92.2)	188 (60.5)
South Asian	14 (6.1)	84 (27.0)
East Asian	1 (0.4)	1 (0.3)
African Caribbean	2 (0.9)	12 (3.9)
Other	1 (0.4)	26 (8.4)
All non-Caucasian combined	18 (7.8)	123 (39.6)
	(*n* = 231)	(*n* = 311)
Serum 25(OH)D level (nmol/L), median [IQR]	45 [27, 72]	43 [27, 60]
	(*n* = 231)	(*n* = 294)
Vitamin D status, *n* (%)		
Replete (>50 nmol/L)	106 (45.9)	110 (37.4)
Insufficient (25–50 nmol/L)	73 (31.6)	125 (42.5)
Deficient (<25 nmol/L)	52 (22.5)	59 (20.1)
	(*n* = 231)	(*n* = 294)
Received cholecalciferol treatment, *n* (%)	116 (61.7)	66 (22.6)
Maintenance treatment, *n* (% of patients on treatment)	55 (47.4)	51 (72.9)
High-dose booster treatment, *n* (%)	61 (52.6)	19 (27.1)
	(*n* = 188)	(*n* = 292)
Positive SARS-NCoV2 swab, *n* (%)	219 (94.8)	306 (98.4)
	(*n* = 231)	(*n* = 311)
Death, *n* (%)	55 (24.3)	64 (22.8)
	(*n* = 226)	(*n* = 281)
Hospital-acquired, *n* (%)	76 (33.6)	27 (9.7)
	(*n* = 226)	(*n* = 277)
Received CPAP, *n* (%)	39 (17.2)	14 (4.5)
	(*n* = 227)	(*n* = 309)
Received IMV, *n* (%)	16 (7.0)	7 (2.3)
	(*n* = 229)	(*n* = 309)
Discharged, *n* (%)	173 (75.2)	249 (80.3)
	(*n* = 230)	(*n* = 310)
Length of stay (days), median [IQR]	15 [8, 25]	7 [3, 15]
	(*n* = 227)	(*n* = 305)

The numbers of patients with available data are indicated in parentheses in each row. Full summary statistics are available in Tables S2 and S3.

**Table 4:** Number of participants from the validation cohort on different regimens of cholecalciferol booster therapy.

Cholecalciferol Regimen	Royal Preston Hospital, *n* (%) [*n* = 59]	UHL, *n* (%) [*n* = 19]
50,000 IU weekly	1 (1.7)	1 (5.3)
40,000 IU weekly	39 (66.1)	1 (5.3)
20,000 IU twice weekly	-	7 (36.8)
5000 IU daily	-	1 (5.3)
4000 IU daily	-	1 (5.3)
20,000 IU weekly	19 (32.2)	-
40,000 IU one-off dose	-	1 (5.3)

All participants received cholecalciferol booster therapy for a maximum of 7 weeks.

**Table 5:** Differences in numbers of participants prescribed cholecalciferol booster therapy by centre.

Centre	Prescribed Booster Therapy, *n* (%)	Total Number of Participants with Information Available Regarding Cholecalciferol Prescription
Tameside General Hospital	73 (17.1)	428
Royal Preston Infirmary	59 (26.0)	227
UHL	19 (6.4)	296
Validation cohort (combined)	78 (14.9)	523

**Table 6:** Univariate analysis of potential predictors associated with mortality from COVID-19 in the primary cohort.

Predictor Variable	OR (95% CI)	*p*-Value (Unadjusted)	OR_adj_ (95% CI)	*p*-Value (Adjusted)	*n*
Age > 74 years	2.71 (1.82–4.04)	<0.001	0.48 (0.24–0.97)	0.040	430
Female sex	1.08 (0.73–1.58)	0.712	1.01 (0.66–1.53)	0.971	430
Hospital-acquired COVID-19	1.39 (0.91–2.12)	0.16	1.23 (0.77–1.95)	0.384	428
Non-Caucasian ethnicity	0.73 (0.41–1.30)	0.288	1.83 (0.90–3.70)	0.093	430
Baseline serum 25(OH)D levels	1.00 (0.99–1.01)	0.429	1.01 (1.00–1.01)	0.290	207
Vitamin D deficiency	0.90 (0.50–1.59)	0.707	0.78 (0.42–1.45)	0.439	207
Treatment with cholecalciferol booster therapy	0.29 (0.15–0.54)	<0.001	0.25 (0.12–0.49)	<0.001	414
Admission SpO2 < 96%	0.94 (0.64–1.39)	0.763	0.84 (0.55–1.29)	0.432	410
Admission CRP > 82 mg/L	1.57 (1.06–2.30)	0.023	1.62 (1.06–2.49)	0.026	421
Admission creatinine > 84 μmol/L	2.02 (1.37–2.97)	<0.001	1.63 (1.04–2.55)	0.032	427
Received CPAP	0.57 (0.28–1.15)	0.116	1.02 (0.48–2.18)	0.955	430
Received IMV	0.71 (0.32–1.55)	0.386	2.09 (0.81–5.39)	0.127	430
Length of stay > 10 days	0.99 (0.97–1.00)	0.168	0.99 (0.97–1.00)	0.144	410
Diabetes (types 1 and 2 combined)	1.21 (0.79–1.83)	0.381	1.01 (0.64–1.59)	0.974	430
Admission glucose > 6.7 mmol/L	1.36 (0.93–2.00)	0.117	1.39 (0.89–2.18)	0.152	414
COPD	1.50 (0.95–2.35)	0.081	1.11 (0.68–1.79)	0.679	430
Asthma	0.23 (0.10–0.50)	<0.001	0.31 (0.13–0.71)	0.006	430
IHD	2.59 (1.55–4.34)	<0.001	1.91 (1.10–3.31)	0.021	430
Current or previous ACS	1.04 (0.56–1.91)	0.906	0.81 (0.42–1.55)	0.519	430
Heart failure	1.46 (0.82–2.60)	0.198	0.99 (0.53–1.85)	0.969	430
Hypertension	1.25 (0.85–1.83)	0.263	0.89 (0.58–1.37)	0.597	430
Current or previous TIA or stroke	1.27 (0.65–2.45)	0.483	1.09 (0.54–2.19)	0.812	430
Dementia	1.66 (0.95–2.92)	0.077	1.00 (0.54–1.83)	0.995	430
Obesity	0.07 (0.01–0.53)	0.010	0.14 (0.02–1.09)	0.061	430
Malignancy of solid organ	1.99 (1.18–3.37)	0.010	1.61 (0.93–2.80)	0.090	430
Malignancy of skin	1.46 (0.36–5.90)	0.598	1.74 (0.38–7.86)	0.474	430
Haematological malignancy	2.45 (0.58–10.39)	0.224	1.69 (0.38–7.52)	0.490	430
Solid organ transplant	4.40 (0.45–42.61)	0.201	5.23 (0.52–53.00)	0.161	430
Inflammatory arthritis	0.47 (0.15–1.48)	0.198	0.40 (0.12–1.38)	0.147	430
Inflammatory bowel disease	0.36 (0.04–3.23)	0.360	0.26 (0.03–2.50)	0.244	430

The primary cohort was recruited from Tameside General Hospital only. In the adjusted analysis, variables have been adjusted for age, sex, obesity, ethnicity, and the presence of diabetes (types 1 and 2 combined). The *n* column refers to the total number of participants included in each analysis (i.e., each row). The *n* differs between variables because of missing data. For the total number of participants with each potential predictor variable studied, please refer back to Table 1.

**Table 7:** Predictors associated with death from COVID-19 in the primary cohort, multivariate analysis, *n* = 203.

Predictor Variable	OR_adj_ (95% CI)	*p*-Value
Age > 74 years	1.72 (0.80–3.70)	0.167
Treatment with cholecalciferol booster therapy	0.13 (0.05–0.35)	<0.001
Admission CRP > 82 mg/L	2.01 (1.03–3.91)	0.040
Admission creatinine > 84 μmol/L	1.53 (0.74–3.16)	0.255
Asthma	0.18 (0.04–0.94)	0.042
IHD	1.76 (0.71–4.34)	0.222
Female sex	1.47 (0.73–2.97)	0.286
Obesity	1	
Diabetes (types 1 and 2 combined)	0.86 (0.41–1.81)	0.689
Non-Caucasian ethnicity	1.61 (0.57–4.54)	0.369
Baseline serum 25(OH)D level	1.00 (0.99–1.01)	0.723

**Table 8:** Univariate analysis of potential predictors associated with mortality from COVID-19 in the validation cohort.

Predictor Variable	OR (95% CI)	*p*-Value (Unadjusted)	OR_adj_ (95% CI)	*p*-Value (Adjusted)	*n*
Age > 73 years	3.53 (2.24–5.55)	<0.001	3.26 (1.99–5.36)	<0.001	492
Female sex	0.78 (0.51–1.18)	0.239	0.68 (0.44–1.06)	0.087	492
Hospital-acquired COVID-19	1.17 (0.71–1.93)	0.547	1.11 (0.65–1.90)	0.703	488
Non-Caucasian ethnicity	0.54 (0.32–0.91)	0.021	1.05 (0.59–1.89)	0.860	492
Baseline serum 25(OH)D levels	1.00 (0.99–1.01)	0.601	1.00 (0.99–1.00)	0.443	477
Vitamin D deficiency	1.32 (0.80–2.17)	0.271	1.87 (1.09–3.27)	0.024	477
Treatment with cholecalciferol booster therapy	0.55 (0.28–1.09)	0.087	0.40 (0.20–0.82)	0.012	474
Admission SpO2 < 96%	2.01 (1.32–3.05)	0.001	1.88 (1.21–2.93)	0.005	489
Admission CRP > 73 mg/L	1.95 (1.28–2.99)	0.002	1.87 (1.19–2.95)	0.007	483
Admission creatinine > 83 μmol/L	2.95 (1.90–4.59)	<0.001	2.10 (1.29–3.42)	0.003	489
Received CPAP	2.02 (1.10–3.70)	0.024	4.48 (2.17–9.22)	<0.001	487
Received IMV	0.87 (0.28–2.67)	0.808	1.76 (0.52–5.96)	0.361	490
Length of stay >11 days	1.63 (1.07–2.47)	0.023	1.32 (0.83–2.08)	0.237	486
Diabetes (types 1 and 2 combined)	0.88 (0.55–1.40)	0.586	0.90 (0.55–1.45)	0.659	492
Admission glucose > 6·9 mmol/L	1.40 (0.90–2.17)	0.136	1.69 (1.02–2.80)	0.040	425
COPD	1.37 (0.75–2.51)	0.305	0.95 (0.50–1.81)	0.877	492
Asthma	0.36 (0.16–0.82)	0.015	0.54 (0.23–1.25)	0.148	492
IHD	2.78 (1.68–4.61)	<0.001	1.85 (1.07–3.18)	0.027	492
Current or previous ACS	1.31 (0.40–4.27)	0.649	1.05 (0.31–3.55)	0.935	492
Heart failure	2.29 (1.28–4.08)	0.005	1.61 (0.87–2.99)	0.128	492
Hypertension	1.45 (0.96–2.22)	0.081	1.06 (0.67–1.68)	0.806	492
Current or previous TIA or stroke	1.61 (0.89–2.89)	0.113	1.18 (0.64–2.18)	0.603	492
Dementia	2.34 (1.39–3.92)	0.001	1.18 (0.67–2.08)	0.573	492
Obesity	0.46 (0.23–0.92)	0.028	0.76 (0.36–1.60)	0.471	492
Malignancy of solid organ	1.55 (0.85–2.82)	0.151	1.13(0.59–2.14)	0.712	492
Malignancy of skin	0.65 (0.08–5.61)	0.695	0.58 (0.06–5.90)	0.645	492
Haematological malignancy	1.53 (0.57–4.12)	0.399	1.09 (0.39–3.06)	0.863	492
Solid organ transplant	0.81 (0.09–7.35)	0.854	2.16 (0.19–24.49)	0.533	492
Inflammatory arthritis	0.32 (0.04–2.53)	0.280	0.22 (0.03–1.81)	0.161	492
Inflammatory bowel disease	0.54 (0.06–4.53)	0.570	0.78 (0.08–7.36)	0.829	492

The validation cohort comprised patients recruited from both Royal Preston Hospital and UHL. In the adjusted analysis, variables have been adjusted for age, sex, obesity, ethnicity, and the presence of diabetes (types 1 and 2 combined). The *n* column refers to the total number of participants included in each analysis (i.e., each row). The *n* differs between variables because of missing data. For the total number of participants with each potential predictor variable studied, please refer back to Table 2. Cut-off values for binary variables differ slightly from the primary cohort, as these are based on median values of each cohort, and not for the total study population.

**Table 9:** Predictors associated with death from COVID-19 in the validation cohort, multivariate analysis, *n* = 449.

Predictor Variable	OR_adj_ (95% CI)	*p*-Value
Age > 73 years	2.90 (1.58–5.33)	0.001
Treatment with cholecalciferol booster therapy	0.38 (0.17–0.84)	0.018
Admission SpO2 < 96%	2.08 (1.22–3.53)	0.007
Admission CRP > 73 mg/L	1.63 (0.98–2.72)	0.060
Creatinine > 83 μmol/L	2.38 (1.39–4.07)	0.002
Received CPAP	2.54 (1.13–5.69)	0.023
IHD	2.51 (1.36–4.64)	0.003
Female	1.11 (0.67–1.86)	0.683
Obesity	0.74 (0.33–1.66)	0.471
Non-Caucasian ethnicity	0.60 (0.30–1.22)	0.159
Diabetes (types 1 and 2 combined)	0.66 (0.37–1.16)	0.147
Baseline serum 25(OH)D	0.99 (0.98–1.00)	0.215
Centre (Preston vs Leicester)	0.66 (0.37–1.18)	0.160

The validation cohort comprised patients recruited from both Royal Preston Hospital and UHL.

## Supplementary Materials

The following are available online at https://www.mdpi.com/2072-6643/12/12/3799/s1, Table S1: Full set of data variables collected for study, Table S2: Participant characteristics of the patient population recruited from Royal Preston Hospital, Table S3: Participant characteristics of the patient population recruited from University Hospitals of Leicester, Table S4: Univariate analysis of potential predictors associated with mortality from COVID-19 in the primary cohort, participants with positive SARS-CoV-2 swab only, Tables S5–S7: Predictors associated with death from COVID-19 in the primary cohort, univariate analysis, sub-analysis by vitamin D status (vitamin D deficiency, vitamin D insufficiency and deficiency combined, and vitamin D replete, respectively), Table S8: Predictors associated with death from COVID-19 in the primary cohort, multivariate analysis, participants with positive SARS-CoV-2 swab only, Table S9: Univariate analysis of potential predictors associated with mortality from COVID-19 in the validation cohort, participants with positive SARS-CoV-2 swab only, Tables S10–S12: Predictors associated with death from COVID-19 in the validation cohort, univariate analysis, sub-analysis by vitamin D status (vitamin D deficiency, vitamin D insufficiency and deficiency combined, and vitamin D replete, respectively), Table S13: Predictors associated with death from COVID-19 in the validation cohort, multivariate analysis, participants with positive SARS-CoV-2 swab only.

## References

[1] Jakovac H. (2020). COVID-19 and vitamin D-Is there a link and an opportunity for intervention?. Am. J. Physiol. Endocrinol. Metab..

[2] Caccialanza R., Laviano A., Lobascio F., Montagna E., Bruno R., Ludovisi S., Corsico A.G., Di Sabatino A., Belliato M., Calvi M. (2020). Early nutritional supplementation in non-critically ill patients hospitalized for the 2019 novel coronavirus disease (COVID-19): Rationale and feasibility of a shared pragmatic protocol. Nutrition.

[3] Carter S.J., Baranauskas M.N., Fly A.D. (2020). Considerations for Obesity, Vitamin D, and Physical Activity Amid the COVID-19 Pandemic. Obesity.

[4] Malaguarnera L. (2020). Vitamin D3 as Potential Treatment Adjuncts for COVID-19. Nutrients.

[5] Teymoori-Rad M., Shokri F., Salimi V., Marashi S.M. (2019). The interplay between vitamin D and viral infections. Rev. Med. Virol..

[6] Grant W.B., Lahore H., McDonnell S.L., Baggerly C.A., French C.B., Aliano J.L., Bhattoa H.P. (2020). Evidence that Vitamin D Supplementation Could Reduce Risk of Influenza and COVID-19 Infections and Deaths. Nutrients.

[7] Calder P.C., Carr A.C., Gombart A.F., Eggersdorfer M. (2020). Optimal Nutritional Status for a Well-Functioning Immune System Is an Important Factor to Protect against Viral Infections. Nutrients.

[8] Silberstein M. (2020). Vitamin D: A simpler alternative to tocilizumab for trial in COVID-19?. Med. Hypotheses.

[9] Panagiotou G., Tee S.A., Ihsan Y., Athar W., Marchitelli G., Kelly D., Boot C.S., Stock N., Macfarlane J., Martineau A.R. (2020). Low serum 25-hydroxyvitamin D (25[OH]D) levels in patients hospitalized with COVID-19 are associated with greater disease severity. Clin. Endocrinol..

[10] Ilie P.C., Stefanescu S., Smith L. (2020). The role of vitamin D in the prevention of coronavirus disease 2019 infection and mortality. Aging Clin. Exp. Res..

[11] Munshi R., Hussein M.H., Toraih E.A., Elshazli R.M., Jardak C., Sultana N., Youssef M.R., Omar M., Attia A.S., Fawzy M.S. (2020). Vitamin D insufficiency as a potential culprit in critical COVID-19 patients. J. Med. Virol..

[12] HRA (2020). Extension of COVID-19 COPI Notice. https://www.hra.nhs.uk/about-us/news-updates/extension-covid-19-copi-notice/.

[13] WHO (2020). International Guidelines for Certification and Classification (Coding) of Covid-19 as Cause of Death. https://www.who.int/classifications/icd/Guidelines_Cause_of_Death_COVID-19-20200420-EN.pdf?ua=1.

[14] GMMMG (2016). Treatment of Vitamin D Deficiency and Insufficiency in Adults. http://gmmmg.nhs.uk/docs/nts/NTS-Recommendation-on-Vitamin-D-deficiency-and-insufficiency-adults.pdf.

[15] RIQAS https://www.randox.com/external-quality-assessment/.

[16] DEQAS http://www.deqas.org/.

[17] UK NEQAS https://ukneqas.org.uk/.

[18] Hastie C.E., Mackay D.F., Ho F., Celis-Morales C.A., Katikireddi S.V., Niedzwiedz C.L., Jani B.D., Welsh P., Mair F.S., Gray S.R. (2020). Vitamin D concentrations and COVID-19 infection in UK Biobank. Diabetes Metab. Syndr..

[19] Rhodes J.M., Subramanian S., Laird E., Kenny R.A. (2020). Editorial: Low population mortality from COVID-19 in countries south of latitude 35 degrees North supports vitamin D as a factor determining severity. Aliment. Pharmacol. Ther..

[20] Merzon E., Tworowski D., Gorohovski A., Vinker S., Golan Cohen A., Green I., Frenkel-Morgenstern M. (2020). Low plasma 25(OH) vitamin D level is associated with increased risk of COVID-19 infection: An Israeli population-based study. FEBS J..

[21] Meltzer D.O., Best T.J., Zhang H., Vokes T., Arora V., Solway J. (2020). Association of Vitamin D Status and Other Clinical Characteristics with COVID-19 Test Results. JAMA Netw. Open.

[22] SACN U (2016). Vitamin D and Health. https://www.gov.uk/government/groups/scientific-advisory-committee-on-nutrition.

[23] Carlberg C., Haq A. (2018). The concept of the personal vitamin D response index. J. Steroid Biochem. Mol. Biol..

[24] Carlberg C., Seuter S., de Mello V.D., Schwab U., Voutilainen S., Pulkki K., Nurmi T., Virtanen J., Tuomainen T.-P., Uusitupa M. (2013). Primary vitamin D target genes allow a categorization of possible benefits of vitamin D supplementation. PLoS ONE.

[25] Seuter S., Virtanen J.K., Nurmi T., Pihlajamäki J., Mursu J., Voutilainen S., Tuomainen T.-P., Neme A., Carlberg C. (2017). Molecular evaluation of vitamin D responsiveness of healthy young adults. J. Steroid Biochem. Mol. Biol..

[26] Castillo M.E., Costa L.M.E., Barrios J.M.V., Díaz J.F.A., Miranda J.L., Bouillon R., Gomez J.M.Q. (2020). Effect of calcifediol treatment and best available therapy versus best available therapy on intensive care unit admission and mortality among patients hospitalized for COVID-19: A pilot randomized clinical study. J. Steroid Biochem. Mol. Biol..

[27] Rastogi A., Bhansali A., Khare N., Suri V., Yaddanapudi N., Sachdeva N., Puri G.D., Malhotra P. (2020). Short term, high-dose vitamin D supplementation for COVID-19 disease: A randomised, placebo-controlled, study (SHADE study). Postgrad. Med. J..

[28] ONS (2020). Deaths Involving COVID-19, England and Wales: Deaths Occurring in June 2020. https://www.ons.gov.uk/peoplepopulationandcommunity/birthsdeathsandmarriages/deaths/bulletins/deathsinvolvingcovid19englandandwales/deathsoccurringinjune2020.

[29] Iaccarino G., Grassi G., Borghi C., Ferri C., Salvetti M., Volpe M. (2020). Age and Multimorbidity Predict Death Among COVID-19 Patients: Results of the SARS-RAS Study of the Italian Society of Hypertension. Hypertension.

