# Supplementary material for: High-Dose Cholecalciferol Booster Therapy is Associated with a Reduced Risk of Mortality in Patients with COVID-19: A Cross-Sectional Multi-Centre Observational Study
Source: Nutrients. 2020 Dec 11;12(12):3799. doi: 10.3390/nu12123799 (PMC7763301; doi:10.3390/nu12123799)
Supplement: Supplementary file 1 [file nutrients-12-03799-s001.pdf]

Supplementary information

Table S1. Full set of data variables collected for study, including sources of data.

	Source	Purpose of collection	Form of data
Patient identifiable data	Electronic patient record (EPR)	Data linkage	String
Diagnosis on admission	Patient medical records	Check meets inclusion criteria	String
Comorbidities	Patient medical records	Analysis of covariates	Binary i·e· has/does not have condition
Weight and height	EPR/patient medical records	Calculation of body mass index (BMI), then analysis of covariate	Continuous
Diabetes (types 1 and 2)	Patient medical records	Analysis of covariate	Binary i·e· yes/no for each type
SARS-CoV-2 swab results	EPR	Analysis of covariate	Binary i·e· positive/negative
Treatment history	Patient medical records	Analysis of outcomes	Binary i·e· received/did not receive
Serum 25-hydroxyvitamin D (25(OH)D) level	EPR/laboratory	Analysis of outcome	Continuous/conversion to categorical variable according to vitamin D status
High-dose cholecalciferol booster therapy	Patient medical records	Analysis of predictor	Binary i·e· yes/no
Ethnicity	EPR/patient medical records	Analysis of predictor	Categorical
Admission peripheral capillary oxygen saturation (SpO_2)	Patient medical records	Analysis of predictor	Continuous
C-reactive protein (CRP) on admission	EPR	Analysis of predictor	Continuous
Creatinine on admission	EPR	Analysis of predictor	Continuous
Adjusted calcium on admission	EPR	Analysis of predictor	Continuous
Death	EPR/patient medical records	Analysis of outcome	Binary i·e· yes/no
Discharge	EPR/patient medical records	Analysis of predictor	Binary i·e· yes/no
Length of stay	EPR/patient medical records	Analysis of predictor	Continuous – could be converted to categorical/binary
Continuous positive airways pressure (CPAP) therapy	EPR/patient medical records	Analysis of predictor	Binary i·e· yes/no
Invasive mechanical ventilation (IMV) therapy	EPR/patient medical records	Analysis of predictor	Binary i·e· yes/no

Full list of comorbidities analysed:

- Chronic obstructive pulmonary disease (COPD).
- Asthma.
- Ischaemic heart disease (IHD).
- Myocardial infarction (MI).
- Heart failure.
- Hypertension.
- Transient ischaemia attack (TIA).
- Stroke.
- Dementia.
- Obesity.
- Solid cancer.
- Skin cancer.

- Haematological malignancy.
- Solid organ transplant.
- Inflammatory bowel disease.
- Inflammatory arthritis.

Table S2. Participant characteristics of the patient population recruited from Royal Preston Hospital.

		Number of participants with available data
Age (years), median [IQR]	76 [61, 84]	231
Female, n (%)	106 (42·2)	231
**Ethnicity, n (%)**<br>Caucasian<br>South Asian<br>East Asian<br>African Caribbean<br>Other<br>All non-Caucasian combined	 213 (92·2)<br>14 (6·1)<br>1 (0·4)<br>2 (0·9)<br>1 (0·4)<br>18 (7·8)	231
Vitamin D level, median [IQR]	45 [27, 72]	231
**Vitamin D status, n (%)**<br>Replete (>50 nmol/L)<br>Insufficient (25-50 nmol/L)<br>Deficient (<25 nmol/L)	 106 (45·9)<br>73 (31·6)<br>52 (22·5)	231
**Received vitamin D treatment, n (%)**<br>Maintenance treatment, n (% of patients on treatment)<br>High-dose booster treatment, n (%)	116 (61·7)<br>55 (47·4)<br>61 (52·6)	231
Positive SARS-NCoV2 swab, n (%)	219 (94·8)	231
Death, n (%)	55 (24·3)	226
Hospital-acquired, n (%)	76 (33·6)	226
Pulmonary embolism (PE) during admission, n (%)	8 (3·5)	229
Oxygen saturation (SpO_2) on admission, median [IQR]	95 [93, 98]	231
C-reactive protein (CRP) on admission (mg/L), median [IQR]	90·5 [32, 166]	230
Creatinine on admission (μmol/L), median [IQR]	86 [65, 129]	231
Adjusted calcium on admission (mmol/L), median [IQR]	2·4 [2·3, 2·5]	214
Random glucose on admission (mmol/L), median [IQR]	6·8 [5·9, 8·4]	224
Received low-flow oxygen (<10L/min), n (%)	169 (75·8)	223
Received high-flow oxygen (≥10L/min), n (%)	72 (31·9)	226
Received CPAP, n (%)	39 (17·2)	227
Received invasive ventilation, n (%)	16 (7·0)	229
Discharged, n (%)	173 (75·2)	230
Length of stay (days), median [IQR]	15 [8, 25]	227
Diabetes mellitus (both types I and II), n (%)	77 (33·6)	229
Chronic obstructive pulmonary disease (COPD), n (%)	39 (16·9)	231
Asthma, n (%)	24 (10·4)	231
Ischaemic heart disease (IHD), n (%)	48 (20·8)	231
Current or previous acute coronary syndrome, n (%)	1 (0·4)	231
Heart failure, n (%)	24 (10·4)	231
Hypertension, n (%)	89 (38·5)	231
Current or previous transient ischaemic attack (TIA) or stroke, n (%)	34 (14·7)	231
Dementia, n (%)	40 (17·3)	231
Obesity, n (%)	16 (6·9)	231
Malignancy of solid organ, n (%)	26 (11·3)	231
Malignancy of skin, n (%)	0 (0)	231
Haematological malignancy, n (%)	12 (5·2)	231
Solid organ transplant, n (%)	1 (0·4)	231
Inflammatory arthritis, n (%)	5 (2·2)	231
Inflammatory bowel disease, n (%)	8 (3·5)	231

Table S3. Participant characteristics of the patient population recruited from University Hospitals of Leicester.

		Number of participants with available data
Age (years), median [IQR]	70 [56, 84]	309
Female, n (%)	145 (57·8)	309
**Ethnicity, n (%)**<br>Caucasian<br>South Asian<br>East Asian<br>African Caribbean<br>Other<br>All non-Caucasian combined	 188 (60·5)<br>84 (27·0)<br>1 (0·3)<br>12 (3·9)<br>26 (8·4)<br>123 (39·6)	311
Vitamin D level, median [IQR]	43 [27, 60]	294
**Vitamin D status, n (%)**<br>Replete (>50 nmol/L)<br>Insufficient (25-50 nmol/L)<br>Deficient (<25 nmol/L)	 110 (37·4)<br>125 (42·5)<br>59 (20·1)	294
**Received vitamin D treatment, n (%)**<br>Maintenance treatment, n (% of patients on treatment)<br>High-dose booster treatment, n (%)	66 (22·6)<br>51 (72·9)<br>19 (27·1)	292
Positive SARS-NCoV2 swab, n (%)	306 (98·4)	311
Death, n (%)	64 (22·8)	281
Hospital-acquired, n (%)	27 (9·7)	277
Pulmonary embolism (PE) during admission, n (%)	9 (2·9)	310
Oxygen saturation (SpO_2) on admission, median [IQR]	96 [95, 98]	306
C-reactive protein (CRP) on admission (mg/L), median [IQR]	66 [23, 133]	297
Creatinine on admission (μmol/L), median [IQR]	82 [66, 106]	307
Adjusted calcium on admission (mmol/L), median [IQR]	2·2 [2·1, 2·3]	184
Random glucose on admission (mmol/L), median [IQR]	7 [5·7, 9·1]	241
Received low-flow oxygen (<10L/min), n (%)	130 (42·2)	308
Received high-flow oxygen (≥10L/min), n (%)		
Received CPAP, n (%)	14 (4·5)	309
Received invasive ventilation, n (%)	7 (2·3)	309
Discharged, n (%)	249 (80·3)	310
Length of stay (days), median [IQR]	7 [3, 15]	305
Diabetes mellitus (both types I and II), n (%)	76 (25·3)	300
Chronic obstructive pulmonary disease (COPD), n (%)	26 (8·4)	311
Asthma, n (%)	46 (14·8)	311
Ischaemic heart disease (IHD), n (%)	36 (11·6)	311
Current or previous acute coronary syndrome, n (%)	13 (4·2)	311
Heart failure, n (%)	34 (10·9)	311
Hypertension, n (%)	103 (33·1)	311
Current or previous transient ischaemic attack (TIA) or stroke, n (%)	27 (8·7)	311
Dementia, n (%)	41 (13·2)	31
Obesity, n (%)	66 (21·2)	311
Malignancy of solid organ, n (%)	34 (10·9)	311
Malignancy of skin, n (%)	6 (1·9)	311
Haematological malignancy, n (%)	8 (2·6)	311
Solid organ transplant, n (%)	4 (1·3)	311
Inflammatory arthritis, n (%)	7 (2·3)	311
Inflammatory bowel disease, n (%)	0 (0)	311

Table S4. Univariate analysis of potential predictors associated with mortality from COVID-19 in the primary cohort, participants with positive SARS-CoV-2 swab only (n = 403).

Predictor variable	*OR (95% CI)*	*p-value (unadjusted)*	*OR_{adj} (95% CI)*	*p-value (adjusted)*	*n*
Age >74 years	2·95 (1·94-4·49)	<0·001	0·56 (0·27-1·17)	0·122	391
Female sex	1·05 (0·71-1·57)	0·799	1·03 (0·66-1·59)	0·905	394
Hospital-acquired	1·29 (0·84-1·99)	0·246	1·14 (0·71-1·84)	0·586	393
Non-Caucasian ethnicity	0·73 (0·40-1·33)	0·303	1·97 (0·94-4·13)	0·075	394
Baseline serum 25(OH)D levels	1·00 (0·99-1·01)	0·489	1·01 (1·00-1·01)	0·311	192
Vitamin D deficiency	0·89 (0·48-1·64)	0·716	0·80 (0·41-1·54)	0·496	192
Treatment with cholecalciferol booster therapy	0·33 (0·17-0·64)	0·001	0·29 (0·14-0·60)	0·001	378
Admission SpO2 <96%	1·26 (0·79-2·00)	0·327	1·44 (0·87-2·40)	0·160	314
Admission CRP >82 mg/L	1·61 (1·07-2·43)	0·022	1·73 (1·11-2·70)	0·016	385
Admission creatinine >84 µmol/L	2·28 (1·50-3·43)	<0·001	1·64 (1·03-2·63)	0·039	391
Received CPAP	0·54 (0·23-1·25)	0·151	1·13 (0·45-2·83)	0·790	394
Received IMV	0·78 (0·33-1·80)	0·556	2·73 (0·94-7·93)	0·065	394
Length of stay >10 days	0·99 (0·65-1·49)	0·944	0·88 (0·56-1·38)	0·572	394
Diabetes (types 1 and 2 combined)	1·20 (0·78-1·87)	0·405	1·04 (0·64-1·68)	0·887	394
Admission glucose >6·7 mmol/L	1·40 (0·93-2·10)	0·109	1·36 (0·85-2·19)	0·205	381
COPD	1·42 (0·88-2·28)	0·155	0·99 (0·60-1·65)	0·978	394
Asthma	0·24 (0·11-0·52)	<0·001	0·33 (0·14-0·77)	0·010	394
IHD	2·62 (1·52-4·49)	<0·001	1·91 (1·08-3·41)	0·027	394
Current or previous ACS	0·93 (0·49-1·75)	0·814	0·70 (0·35-1·39)	0·307	394
Heart failure	1·78 (0·97-3·27)	0·062	1·20 (0·62-2·32)	0·585	394
Hypertension	1·23 (0·82-1·84)	0·314	0·89 (0·57-1·39)	0·610	394
Current or previous TIA or stroke	1·45 (0·73-2·88)	0·287	1·29 (0·62-2·68)	0·502	394
Dementia	1·76 (0·99-3·14)	0·055	1·07 (0·57-2·00)	0·831	394
Obesity	0·08 (0·01-0·58)	0·013	0·16 (0·02-1·22)	0·077	394
Malignancy of solid organ	2·04 (1·18-3·53)	0·011	1·66 (0·93-2·95)	0·086	394
Malignancy of skin	1·41 (0·28-7·07)	0·676	1·29 (0·24-6·86)	0·766	394
Haematological malignancy	2·38 (0·56-10·10)	0·240	1·64 (0·37-7·31)	0·515	394
Solid organ transplant	2·83 (0·25-31·44)	0·398	2·84 (0·24-34·09)	0·410	394
Inflammatory arthritis	0·50 (0·16-1·59)	0·239	0·43 (0·12-1·51)	0·189	394
Inflammatory bowel disease	0·46 (0·05-4·50)	0·508	0·32 (0·03-3·40)	0·344	394

Table S5. Predictors associated with death from COVID-19 in the primary cohort, univariate analysis, participants with vitamin D deficiency only (n = 87).

Predictor variable	*OR (95% CI)*	*p-value (unadjusted)*	*OR_{adj} (95% CI)*	*p-value (adjusted)*	*n*
Age >74 years	3·07 (1·15-8·20)	0·025	2·48 (0·84-7·34)	0·101	82
Female sex	0·77 (0·29-2·02)	0·600	0·42 (0·13-1·32)	0·138	83
Hospital-acquired	2·14 (0·82-5·62)	0·122	1·59 (0·54-4·70)	0·398	82
Non-Caucasian ethnicity	0·24 (0·05-1·14)	0·073	0·73 (0·11-4·68)	0·742	83
Baseline serum 25(OH)D levels	0·95 (0·89-1·02)	0·165	0·97 (0·89-1·04)	0·374	83
Treatment with cholecalciferol booster therapy	0·09 (0·03-0·35)	<0·001	0·05 (0·01-0·22)	<0·001	81
Admission SpO2 <96%	0·75 (0·26-2·18)	0·592	0·76 (0·23-2·50)	0·648	69
Admission CRP >82 mg/L	2·33 (0·87-6·26)	0·092	2·26 (0·72-7·09)	0·162	81
Admission creatinine >84 µmol/L	1·86 (0·72-4·85)	0·203	0·85 (0·25-2·82)	0·785	83
Received CPAP	0·65 (0·16-2·64)	0·549	1·87 (0·32-10·79)	0·483	83
Received IMV	0·27 (0·03-2·31)	0·231	1·33 (0·09-19·93)	0·838	83
Length of stay >10 days	0·85 (0·32-2·25)	0·744	0·56 (0·17-1·81)	0·330	78
Diabetes (types 1 and 2 combined)	3·15 (0·55-4·09)	0·428	2·24 (0·70-7·13)	0·173	83
Admission glucose >6·7 mmol/L	0·93 (0·37-2·35)	0·875	1·42 (0·42-4·74)	0·571	81
COPD	1·72 (0·60-4·96)	0·313	0·87 (0·27-2·84)	0·816	83
Asthma	0·20 (0·02-1·67)	0·138	0·37 (0·04-3·56)	0·391	83
IHD	2·32 (0·61-8·82)	0·218	1·68 (0·38-7·43)	0·492	83
Current or previous ACS	1·28 (0·28-5·78)	0·753	0·98 (0·18-5·32)	0·979	83
Heart failure	0·51 (0·13-2·01)	0·337	0·32 (0·07-1·51)	0·149	83
Hypertension	0·84 (0·33-2·17)	0·725	0·43 (0·14-1·30)	0·135	83
Current or previous TIA or stroke	2·26 (0·52-9·84)	0·277	2·26 (0·40-12·63)	0·354	83
Dementia	0·56 (0·11-2·90)	0·489	0·41 (0·07-2·37)	0·318	83
Obesity	1		1		79
Malignancy of solid organ	4·90 (1·55-15·51)	0·007	2·77 (0·78-0·84)	0·116	83
Malignancy of skin	1		1		83
Haematological malignancy	1		1		82
Solid organ transplant	4·40 (0·38-50·81)	0·235	5·81 (0·44-76·64)	0·181	83
Inflammatory arthritis	1		1		81
Inflammatory bowel disease	2·12 (0·13-35·16)	0·601	5·28 (0·24-111·59)	0·289	83

Table S6. Predictors associated with death from COVID-19 in the primary cohort, univariate analysis, participants with vitamin D deficiency and insufficiency combined only (n = 167).

Predictor variable	*OR (95% CI)*	*p-value (unadjusted)*	*OR_{adj} (95% CI)*	*p-value (adjusted)*	*n*
Age >74 years	1·97 (0·99-3·91)	0·054	1·56 (0·74-3·28)	0·240	159
Female sex	1·02 (0·52-2·02)	0·952	0·80 (0·39-1·68)	0·562	160
Hospital-acquired	1·30 (0·63-2·66)	0·479	1·02 (0·47-2·21)	0·963	159
Non-Caucasian ethnicity	0·59 (0·20-1·68)	0·322	1·54 (0·44-5·39)	0·496	160
Baseline serum 25(OH)D levels	0·99 (0·97-1·02)	0·640	1·00 (0·97-1·03)	0·980	160
Treatment with cholecalciferol booster therapy	0·10 (0·03-0·30)	<0·001	0·07 (0·02-0·24)	<0·001	156
Admission SpO2 <96%	1·49 (0·72-3·07)	0·284	1·52 (0·70-3·29)	0·293	136
Admission CRP >82 mg/L	1·93 (0·95-3·91)	0·069	2·34 (1·08-5·05)	0·031	158
Admission creatinine >84 µmol/L	2·10 (1·05-4·19)	0·035	1·57 (0·71-3·47)	0·268	160
Received CPAP	0·46 (0·16-1·30)	0·142	0·76 (0·25-2·32)	0·627	160
Received IMV	0·53 (0·17-1·67)		1·88 (0·47-7·49)	0·369	160
Length of stay >10 days	0·78 (0·38-1·59)	0·489	0·73 (0·34-1·58)	0·419	146
Diabetes (types 1 and 2 combined)	0·80 (0·37-1·73)	0·571	0·77 (0·33-1·75)	0·527	160
Admission glucose >6·7 mmol/L	0·31 (0·41-1·60)	0·542	1·04 (0·46-2·34)	0·921	154
COPD	0·88 (0·39-2·01)	0·766	0·54 (0·22-1·31)	0·172	160
Asthma	0·35 (0·08-1·63)	0·182	0·43 (0·09-2·13)	0·299	160
IHD	2·33 (0·92-5·93)	0·075	1·60 (0·61-4·19)	0·341	160
Current or previous ACS	1·41 (0·48-4·12)	0·531	1·54 (0·36-3·67)	0·807	160
Heart failure	0·52 (0·18-1·47)	0·216	0·35 (0·12-1·06)	0·064	160
Hypertension	0·76 (0·38-1·52)	0·441	0·65 (0·31-1·39)	0·264	160
Current or previous TIA or stroke	2·44 (0·75-7·99)	0·140	2·55 (0·68-9·47)	0·163	160
Dementia	0·54 (0·14-2·00)	0·355	0·33 (0·08-1·31)	0·115	160
Obesity	1		1		149
Malignancy of solid organ	2·72 (1·15-6·42)	0·022	1·91 (0·77-4·75)	0·165	160
Malignancy of skin	2·29 (0·14-37·40)	0·561	2·16 (0·12-39·42)	0·605	160
Haematological malignancy	2·29 (0·14-37·40)	0·561	2·13 (0·12-38·65)	0·609	160
Solid organ transplant	4·68 (0·41-52·88)	0·212	5·59 (0·47-67·06)	0·175	160
Inflammatory arthritis	1		1		155
Inflammatory bowel disease	2·29 (0·14-37·40)	0·561	2·97 (0·16-55·01)	0·465	160

Table S7. Predictors associated with death from COVID-19 in the primary cohort, univariate analysis, participants who were vitamin D replete only (n = 63).

Predictor variable	*OR (95% CI)*	*p-value (unadjusted)*	*OR_{adj} (95% CI)*	*p-value (adjusted)*	*n*
Age >74 years	3·10 (1·08-8·88)	0·035	2·96 (0·90-9·76)	0·074	62
Female sex	1·16 (0·43-3·16)	0·770	0·96 (0·32-2·91)	0·940	63
Hospital-acquired	3·31 (1·08-10·17)	0·036	3·94 (1·15-13·51)	0·029	63
Non-Caucasian ethnicity	0·61 (0·16-2·28)	0·461	1·45 (0·26-8·18)	0·672	63
Baseline serum 25(OH)D levels	0·99 (0·97-1·01)	0·332	0·99 (0·97-1·01)	0·545	63
Treatment with cholecalciferol booster therapy	0·64 (0·11-3·78)	0·622	1·12 (0·15-8·27)	0·915	63
Admission SpO2 <96%	1·82 (0·58-5·71)	0·306	5·18 (1·11-24·31)	0·037	51
Admission CRP >82 mg/L	1·91 (0·69-5·30)	0·215	1·65 (0·55-4·98)	0·375	62
Admission creatinine >84 μmol/L	1·17 (0·42-3·21)	0·765	0·70 (0·18-2·69)	0·606	62
Received CPAP	1·39 (0·31-6·15)	0·663	2·75 (0·48-15·90)	0·258	63
Received IMV	2·96 (0·50-17·50)	0·232	9·51 (0·83-109·24)	0·071	63
Length of stay >10 days	2·13 (0·76-6·01)	0·152	2·07 (0·67-6·41)	0·208	61
Diabetes (types 1 and 2 combined)	0·7 (0·23-2·11)	0·527	0·62 (0·18-2·06)	0·431	63
Admission glucose >6·7 mmol/L	1·35 (0·48-3·78)	0·571	1·92 (0·61-5·98)	0·264	61
COPD	2·07 (0·66-6·54)	0·214	1·74 (0·53-5·79)	0·363	63
Asthma	1		1		55
IHD	1·77 (0·48-6·56)	0·392	2·65 (0·46-15·24)	0·276	63
Current or previous ACS	2·96 (0·50-17·50)	0·232	10·38 (0·88-121·95)	0·063	63
Heart failure	3·86 (0·69-21·69)	0·125	2·40 (0·39-14·66)	0·343	63
Hypertension	0·96 (0·35-2·62)	0·942	0·68 (0·20-2·29)	0·535	63
Current or previous TIA or stroke	0·24 (0·03-2·17)	0·203	0·21 (0·02-2·07)	0·183	63
Dementia	1·36 (0·18-10·32)	0·766	1·00 (0·12-8·34)	0·999	63
Obesity	1		1		60
Malignancy of solid organ	0·72 (0·19-2·76)	0·633	0·63 (0·15-2·67)	0·535	63
Malignancy of skin	1·35 (0·08-22·54)	0·836	1·45 (0·08-25·92)	0·803	63
Haematological malignancy	1·36 (0·18-10·32)	0·766	0·91 (0·10-8·29)	0·936	63
Solid organ transplant	1		1		63
Inflammatory arthritis	0·42 (0·05-4·31)	0·468	0·28 (0·02-3·34)	0·318	63
Inflammatory bowel disease	1		1		62

Table S8. Predictors associated with death from COVID-19 in the primary cohort, multivariate analysis, participants with positive SARS-CoV-2 swab only (n = 169).

Predictor variable	*OR_{adj} (95% CI)*	*p-value*
Treatment with cholecalciferol booster therapy	0·15 (0·05-0·43)	<0·001
Admission CRP >82 mg/L	2·03 (1·00-4·12)	0·049
Admission creatinine >84μmol/L	1·42 (0·64-3·18)	0·392
Asthma	0·21 (0·04-1·11)	0·066
IHD	1·33 (0·51-3·50)	0·563
Age >74 years	1·91 (0·82-4·42)	0·131
Female sex	1·45 (0·68-3·08)	0·335
Obesity	1	
Non-Caucasian ethnicity	1·60 (0·52-4·86)	0·410
Diabetes (types 1 and 2 combined)	0·86 (0·38-1·94)	0·714
Baseline serum 25(OH)D levels	1·00 (0·99-1·01)	0·924

Table S9. Univariate analysis of potential predictors associated with mortality from COVID-19 in the validation cohort, participants with positive SARS-CoV-2 swab only (n = 525).

Predictor variable	*OR (95% CI)*	*p-value (unadjusted)*	*OR_{adj} (95% CI)*	*p-value (adjusted)*	*n*
Age >74 years	3·23 (2·04-5·09)	<0·001	2·97 (1·79-4·91)	<0·001	489
Female sex	0·86 (0·58-1·32)	0·499	0·75 (0·48-1·18)	0·215	489
Hospital-acquired	1·19 (0·72-1·97)	0·501	1·16 (0·67-1·99)	0·595	486
Non-Caucasian ethnicity	0·56 (0·33-0·95)	0·031	1·11 (0·62-2·00)	0·723	491
Baseline serum 25(OH)D levels	1·00 (0·99-1·01)	0·747	1·00 (0·99-1·00)	0·347	476
Vitamin D deficiency	1·32 (0·80-2·18)	0·272	1·89 (1·09-3·28)	0·023	476
Treatment with cholecalciferol booster therapy	0·56 (0·28-1·11)	0·096	0·42 (0·20-0·85)	0·015	474
Admission SpO2 <96%	1·96 (1·28-3·00)	0·002	1·83 (1·17-2·88)	0·009	487
Admission CRP >82 mg/L	1·93 (125-2·98)	0·003	1·84 (1·16-2·91)	0·010	479
Admission creatinine >84 µmol/L	2·95 (1·88-4·64)	<0·001	2·19 (1·33-3·61)	0·002	487
Received CPAP	2·2 (1·23-4·23	0·009	5·14 (2·45-10·77)	<0·001	486
Received IMV	1·02 (0·33-3·21)	0·967	2·12 (0·61-7·32)	0·237	489
Length of stay >10 days	1·62 (1·06-2·48)	0·027	1·33 (0·84-2·12)	0·228	484
Diabetes (types 1 and 2 combined)	0·88 (0·55-1·41)	0·591	0·92 (0·56-1·49)	0·724	478
Admission glucose >6·7 mmol/L	1·31 (0·84-2·05)	0·233	1·57 (0·94-2·61)	0·084	420
COPD	1·24 (0·66-2·34)	0·502	0·85 (0·43-1·67)	0·638	491
Asthma	0·32 (0·13-0·76)	0·010	0·46 (0·19-1·13)	0·090	491
IHD	2·81 (1·68-4·68)	<0·001	1·92 (1·11-3·32)	0·021	491
Current or previous ACS	1·33 (0·41-4·34)	0·631	1·07 (0·32-3·60)	0·915	491
Heart failure	2·21 (1·23-3·97)	0·008	1·58 (0·84-2·94)	0·153	491
Hypertension	1·50 (0·98-2·30)	0·063	1·11 (0·69-1·76)	0·671	491
Current or previous TIA or stroke	1·44 (0·78-2·64)	0·244	1·07 (0·56-2·01)	0·842	491
Dementia	2·30 (1·42-4·03)	0·001	1·24 (0·70-2·20)	0·454	491
Obesity	0·47 (0·23-0·95)	0·035	0·78 (0·37-1·64)	0·506	491
Malignancy of solid organ	1·63 (0·89-2·97)	0·114	1·20 (0·63-2·27)	0·583	491
Malignancy of skin	0·66 (0·08-5·69)	0·704	0·62 (0·06-6·26)	0·686	491
Haematological malignancy	1·56 (0·58-4·19)	0·382	1·17 (0·42-3·26)	0·768	491
Solid organ transplant	0·83 (0·09-7·46)	0·864	2·17 (0·19-24·50)	0·530	491
Inflammatory arthritis	0·36 (0·05-2·89)	0·337	0·27 (0·03-2·21)	0·222	491
Inflammatory bowel disease	0·55 (0·07-4·59)	0·579	0·82 (0·09-7·62)	0·859	491

Table S10. Predictors associated with death from COVID-19 in the validation cohort, univariate analysis, participants with vitamin D deficiency only (n = 111).

Predictor variable	*OR (95% CI)*	*p-value (unadjusted)*	*OR_{adj} (95% CI)*	*p-value (adjusted)*	*n*
Age >74 years	3·09 (1·27-7·55)	0·013	2·22 (0·81-6·09)	0·122	106
Female sex	1·10 (0·46-2·60)	0·835	0·88 (0·34-2·26)	0·786	106
Hospital-acquired	1·61 (0·62-4·16)	0·330	1·15 (0·40-3·30)	0·790	107
Non-Caucasian ethnicity	0·32 (0·10-1·02)	0·054	0·44 (0·13-1·55)	0·204	107
Baseline serum 25(OH)D levels	1·02 (0·89-1·16)	0·764	1·10 (0·94-1·28)	0·241	107
Treatment with cholecalciferol booster therapy	0·58 (0·21-1·62)	0·300	0·21 (0·06-0·71)	0·012	106
Admission SpO2 <96%	2·04 (0·85-4·90)	0·111	1·73 (0·66-4·55)	0·265	107
Admission CRP >82 mg/L	1·44 (0·60-3·45)	0·411	1·25 (0·48-3·21)	0·648	105
Admission creatinine >84 μmol/L	2·84 (1·11-7·24)	0·029	2·18 (0·75-6·29)	0·151	106
Received CPAP	2·73 (0·83-8·97)	0·099	12·11 (2·25-65·07)	0·004	105
Received IMV	4·62 (0·73-29·24)	0·104	14·71 (1·58-136·63)	0·018	107
Length of stay >10 days	3·17 (1·20-8·39)	0·020	2·34 (0·75-7·24)	0·141	104
Diabetes (types 1 and 2 combined)	1·33 (0·55-3·23)	0·524	1·48 (0·57-3·85)	0·419	103
Admission glucose >6·7 mmol/L	0·87 (0·35-2·15)	0·759	0·73 (0·25-2·13)	0·568	94
COPD	1·03 (0·30-3·54)	0·962	0·46 (0·12-1·73)	0·249	107
Asthma	1		1		100
IHD	1·69 (0·51-5·56)	0·387	0·78 (0·21-2·98)	0·719	107
Current or previous ACS	1		1		105
Heart failure	1·14 (0·21-6·23)	0·881	0·69 (0·11-4·27)	0·694	107
Hypertension	2·81 (1·16-6·82)	0·022	1·76 (0·61-5·08)	0·294	107
Current or previous TIA or stroke	·26 (0·03-2·09)	0·204	0·20 (0·02-1·72)	0·141	107
Dementia	4·20 (1·43-12·36)	0·009	2·12 (0·60-7·49)	0·245	107
Obesity	0·31 (0·09-1·13)	0·077	0·41 (0·10-1·67)	0·214	107
Malignancy of solid organ	2·03 (0·53-7·80)	0·303	1·76 (0·40-7·78)	0·454	107
Malignancy of skin	1		1		106
Haematological malignancy	1·43 (0·12-16·36)	0·776	0·42 (0·03-6·13)	0·526	107
Solid organ transplant	1		1		105
Inflammatory arthritis	1		1		107
Inflammatory bowel disease	1		1		106

Table S11. Predictors associated with death from COVID-19 in the validation cohort, univariate analysis, participants with vitamin D deficiency and insufficiency combined only (n = 309).

Predictor variable	*OR (95% CI)*	*p-value (unadjusted)*	*OR_{adj} (95% CI)*	*p-value (adjusted)*	*n*
Age >74 years	4·18 (2·25-7·78)	<0·001	3·89 (1·95-7·79)	<0·001	286
Female sex	1·02 (0·58-1·81)	0·937	0·84 (0·46-1·56)	0·589	286
Hospital-acquired	1·11 (0·55-2·22)	0·773	0·99 (0·46-2·12)	0·982	285
Non-Caucasian ethnicity	0·44 (0·21-0·91)	0·027	0·88 (0·39-2·00)	0·757	287
Baseline serum 25(OH)D levels	0·98 (0·95-1·00)	0·088	0·97 (0·94-1·00)	0·032	287
Treatment with cholecalciferol booster therapy	0·70 (0·34-1·45)	0·341	0·43 (0·20-0·93)	0·032	283
Admission SpO2 <96%	1·69 (0·95-3·01)	0·076	1·46 (0·79-2·71)	0·230	286
Admission CRP >82 mg/L	1·41 (0·79-2·52)	0·245	1·20 (0·64-2·24)	0·573	277
Admission creatinine >84 µmol/L	3·09 (1·66-5·75)	<0·001	2·08 (1·03-4·21)	0·041	284
Received CPAP	1·44 (0·63-3·29)	0·384	3·31 (1·25-8·78)	0·016	285
Received IMV	1·27 (0·40-4·10)	0·685	2·96 (0·79-11·10)	0·107	286
Length of stay >10 days	2·13 (1·17-3·86)	0·013	1·55 (0·79-3·03)	0·201	281
Diabetes (types 1 and 2 combined)	1·06 (0·58-1·92)	0·861	1·19 (0·62-2·25)	0·601	279
Admission glucose >6·7 mmol/L	1·12 (0·61-2·05)	0·718	1·25 (0·61-2·55)	0·537	248
COPD	1·55 (0·65-3·72)	0·322	1·13 (0·45-2·80)	0·797	287
Asthma	0·17 (0·04-0·72)	0·016	0·21 (0·05-0·96)	0·044	287
IHD	3·11 (1·55-6·24)	0·001	1·80 (0·85-3·80)	0·123	287
Current or previous ACS	0·61 (0·07-5·17)	0·651	0·36 (0·04-3·16)	0·360	287
Heart failure	1·64 (0·68-3·96)	0·268	1·07 (0·42-2·77)	0·881	287
Hypertension	2·12 (1·20-3·77)	0·010	1·35 (0·72-2·53)	0·345	287
Current or previous TIA or stroke	0·96 (0·38-2·48)	0·938	0·60 (0·22-1·61)	0·312	287
Dementia	2·86 (1·41-5·79)	0·004	1·28 (0·59-2·78)	0·537	287
Obesity	0·59 (0·26-1·33)	0·203	0·98 (0·41-2·38)	0·971	287
Malignancy of solid organ	2·29 (0·91-5·73)	0·078	1·74 (0·66-4·60)	0·267	287
Malignancy of skin	1·24 (0·13-12·12)	0·854	2·32 (0·19-28·93)	0·514	287
Haematological malignancy	1·89 (0·46-7·81)	0·376	1·31 (0·29-5·98)	0·728	287
Solid organ transplant	1·24 (0·13-12·12)	0·854	3·41 (0·28-41·11)	0·334	287
Inflammatory arthritis	1·87 (0·17-20·94)	0·613	1·70 (0·14-20·12)	0·675	287
Inflammatory bowel disease	1		1		283

Table S12. Predictors associated with death from COVID-19 in the validation cohort, univariate analysis, participants who were vitamin D replete only (n = 216).

Predictor variable	*OR (95% CI)*	*p-value (unadjusted)*	*OR_{adj} (95% CI)*	*p-value (adjusted)*	*n*
Age >74 years	2·74 (1·33-5·66)	0·006	2·70 (1·24-5·90)	0·012	203
Female sex	0·62 (0·32-1·19)	0·152	0·58 (0·29-1·17)	0·131	203
Hospital-acquired	1·52 (0·72-3·23)	0·273	1·57 (0·70-3·51)	0·276	201
Non-Caucasian ethnicity	0·59 (0·24-1·42)	0·236	1·04 (0·39-2·75)	0·942	203
Baseline serum 25(OH)D levels	1·01 (0·99-1·02)	0·313	1·00 (0·99-1·02)	0·768	203
Treatment with cholecalciferol booster therapy	1		1		188
Admission SpO2 <96%	2·01 (1·04-3·87)	0·037	2·22 (1·10-4·49)	0·026	202
Admission CRP >82 mg/L	2·57 (1·30-5·07)	0·007	3·12 (1·48-6·58)	0·003	201
Admission creatinine >84 µmol/L	2·71 (1·38-5·31)	0·004	2·12 (1·01-4·45)	0·047	202
Received CPAP	3·67 (1·36-9·86)	0·010	9·29 (2·67-32·32)	<0·001	200
Received IMV	1		1		200
Length of stay >10 days	1·54 (0·80-2·96)	0·193	1·40 (0·69-2·82)	0·347	202
Diabetes (types 1 and 2 combined)	0·44 (0·17-1·12)	0·083	0·38 (0·15-0·99)	0·047	199
Admission glucose >6·7 mmol/L	1·66 (0·83-3·30)	0·151	2·25 (1·03-4·91)	0·041	175
COPD	1·40 (0·59-3·28)	0·445	0·91 (0·36-2·31)	0·840	203
Asthma	0·58 (0·16-2·08)	0·402	0·81 (0·21-3·17)	0·765	203
IHD	2·78 (1·27-6·06)	0·010	2·39 (1·01-5·67)	0·047	203
Current or previous ACS	3·38 (0·66-17·32)	0·144	4·97 (0·76-32·32)	0·093	203
Heart failure	3·23 (1·42-7·33)	0·005	3·30 (1·29-8·44)	0·013	203
Hypertension	1·22 (0·62-2·40)	0·557	1·04 (0·50-2·16)	0·910	203
Current or previous TIA or stroke	3·02 (1·34-6·79)	0·008	2·54 (1·08-5·93)	0·032	203
Dementia	2·25 (1·03-4·90)	0·042	1·37 (0·57-3·27)	0·484	203
Obesity	0·35 (0·08-1·59)	0·174	0·60 (0·12-2·95)	0·532	203
Malignancy of solid organ	1·04 (0·44-2·49)	0·930	0·73 (0·28-1·92)	0·527	203
Malignancy of skin	1		1		201
Haematological malignancy	1·41 (0·35-5·68)	0·629	1·03 (0·25-4·34)	0·964	203
Solid organ transplant	1		1		203
Inflammatory arthritis	1		1		195
Inflammatory bowel disease	1·63 (0·14-18·35)		2·30 (0·17-30·79)	0·528	203

Table S13. Predictors associated with death from COVID-19 in the validation cohort, multivariate analysis, participants with positive SARS-CoV-2 swab only (n = 169).

Predictor variable	*OR_{adj} (95% CI)*	*p-value*
Age >73 years	2·58 (1·39-4·79)	0·003
Treatment with cholecalciferol booster therapy	0·38 (0·17-0·84)	0·018
Admission SpO_2 <96%	2·09 (1·22-3·59)	0·007
Admission CRP >73 mg/L	1·70 (1·01-2·86)	0·046
Admission creatinine >83 µmol/L	2·58 (1·48-4·50)	0·001
Received CPAP	2·95 (1·29-6·71)	0·010
IHD	2·77 (1·48-5·17)	0·001
Female sex	1·33 (0·78-2·25)	0·292
Obesity	0·72 (0·32-1·62)	0·429
Non-Caucasian ethnicity	0·58 (0·28-1·19)	0·140
Diabetes (types 1 and 2 combined)	0·65 (0·37-1·16)	0·146
Baseline serum 25(OH)D levels	0·99 (0·98-1·00)	0·149
Centre	0·65 (0·36-1·18)	0·157
